# Colorectal intussusception due to adenocarcinoma presenting as acute bowel obstruction in an adult

**DOI:** 10.1016/j.radcr.2021.02.021

**Published:** 2021-02-26

**Authors:** Alexis Roditis, Salma Jendoubi, Pierre Platevoet, Adrien Le Fouler, Nicolas Sellier

**Affiliations:** a Department of Radiology, Hôpital Jean Verdier, APHP, Bondy, France; b Department of General Surgery, Hôpital Avicenne, APHP, Bobigny, France

**Keywords:** Intussusception, Invagination, Occlusive syndrome, Colorectal adenocarcinoma

## Abstract

Colorectal intussusception is a rare entity in adults presenting an acute abdomen. The authors present a case of a 73-year-old female who presented with an acute large bowel obstruction. Abdominal computed tomography (CT) scan reveals a colorectal intussusception with a colonic distension upstream. Laparoscopy founds out a stenotic tumor on colorectal junction corresponding with an adenocarcinoma on histopathological exam. CT scan is the most specific diagnostic test for intussusception and is superior to ultrasonography and endoscopy and thus should be performed preferentially.

## Introduction

Intussusceptions are a frequent cause of acute abdomen in pediatrics mostly with the ileocecal invagination. It is much uncommon in adults and it can present acutely, subacutely or with a chronic history, most often with occlusive syndromes [1].

In more than one case over 2, it is caused by an organic lesion acting as a lead point mainly lipoma, polyp, adenocarcinoma, and lymphoma [2].

It can rarely occur in the colon, where yet there is no peristalsis, mostly caused by colo-rectal adenocarcinoma. We can find in the literature rarer causes like lipoma, angiolipoma [3]. Because of the predominant share of malignant etiologies in symptomatic intussusceptions among adults, both small bowel, and colonic invagination are usually treated by surgical resection [4].

## Case summary

A 73-year-old female with a history of proctorrhagia for 1 month and a non-honored colposcopy appointment presented to the emergency department for a vomiting described as black-colored and abdominal pain mainly in iliac fossae. On physical examination there was no abdominal defense, but tenderness on left iliac fossa palpation. Vital signs were good and there was no inflammatory syndrome. She had never had abdominal surgery.

**Fig. 1:**
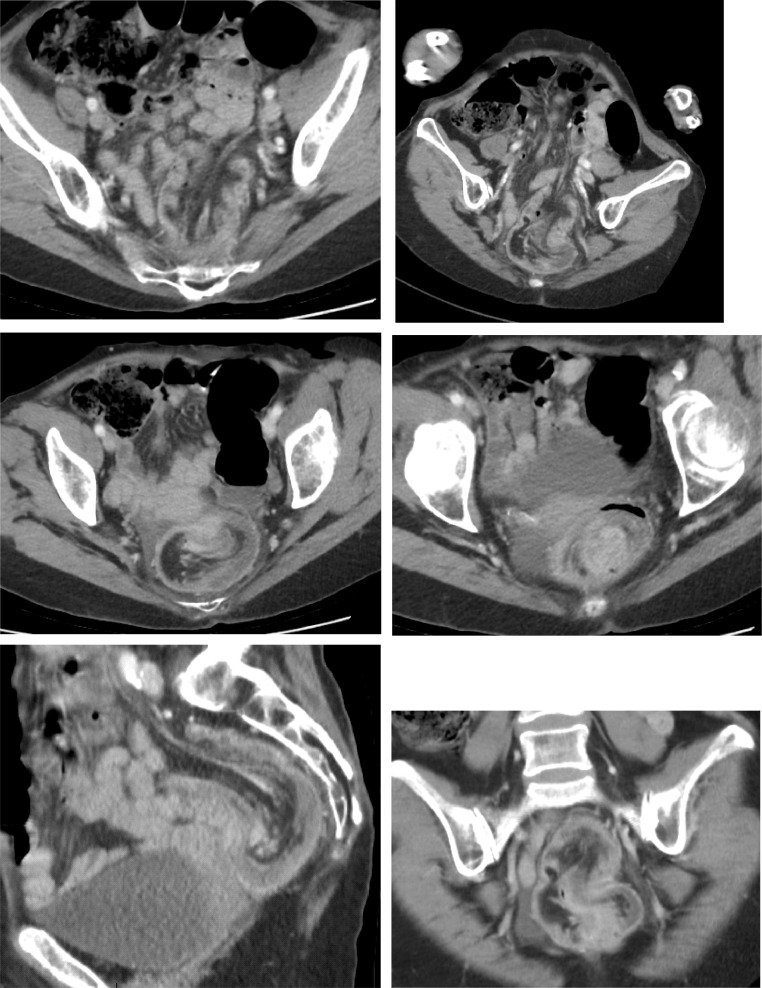
CT scan with axial, sagittal and coronal views respectively demonstrating intussusception of the sigmoid colon into the rectum.

An abdominal computed tomography (CT) scan was performed. It showed typical images of bowel invagination as an inhomogeneous “target” or “sausage”- shaped soft- tissue mass due to the telescoping of the sigmoid colon in the upper rectum with its mesentery (Fig. 1).

**Fig. 2:**
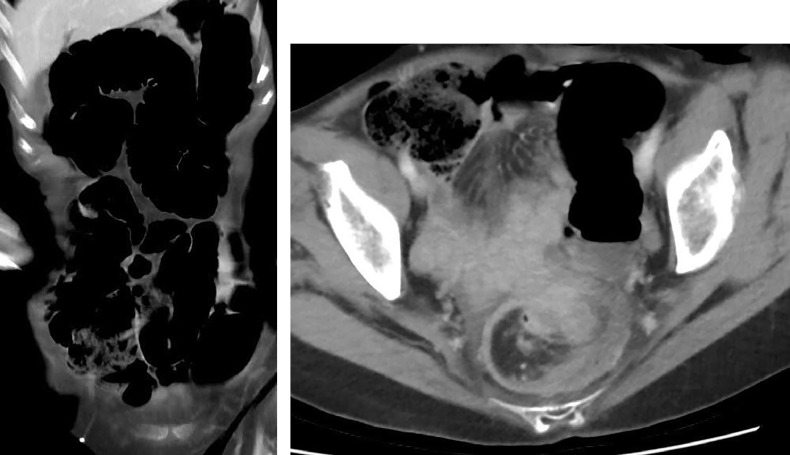
CT scan with coronal and axial views and thick cuts showing large bowel dilation.

Upstream, a colonic distension with an ileocecal valve continent (Fig. 2). There was bowel wall edema and no evidence of either perforation or collection nearby. There was no obvious cause found on the CT-scan.

**Fig. 3:**
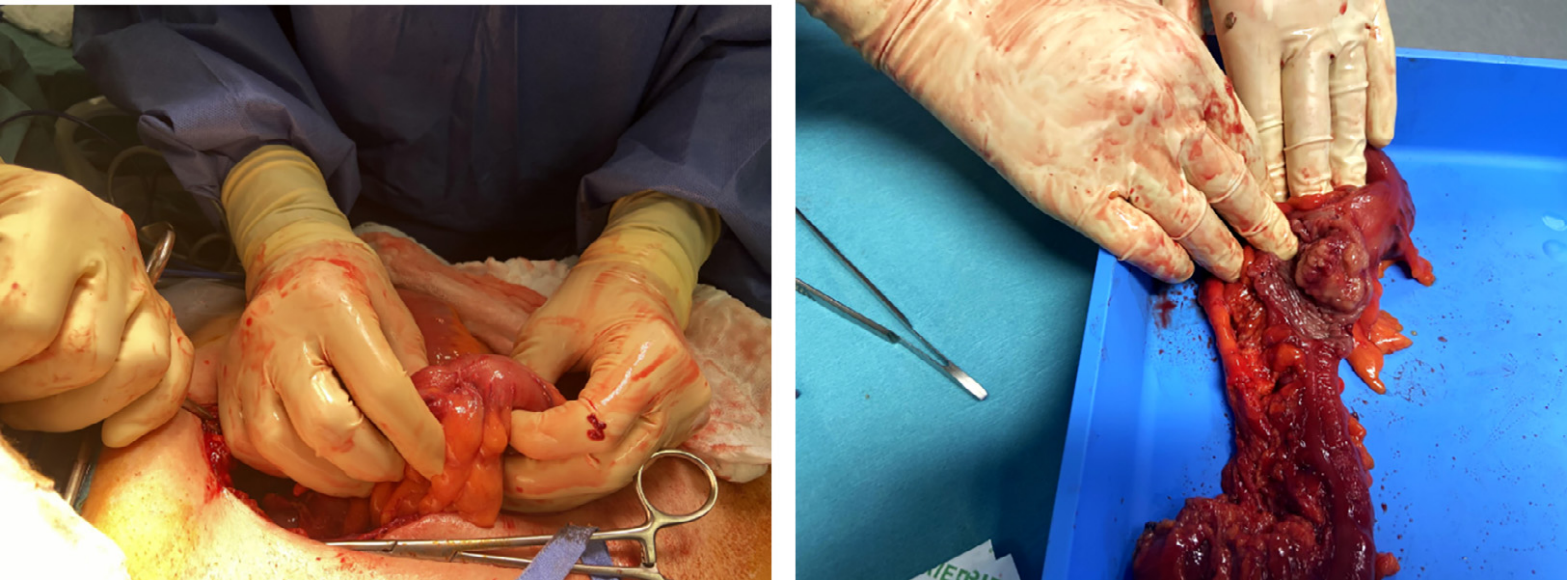
On the left, surgeon showing the bowel stenosis. On the right, after sigmoid resection, surgeon showing the tumor dissected in the lumen of the sigmoid.

The patient was transferred to the surgery department where she underwent a laparoscopy. In the first finding, there was a large colonic distension but no sign of colic ischemia or perforation. Rapidly a laparotomy was performed. A stenosing tumor was found at the rectosigmoid junction after the invagination was reduced (Fig. 3). Hartmann's procedure was performed after resection of the sigmoid colon.

Histopathological examination showed an ulcerative semicircumferential tumor measuring 4 by 3 cm centered on the rectosigmoid junction, corresponding to an adenocarcinoma not otherwise specified invading the muscularis (T2 of TNM colorectal cancer classification).

## Discussion

Colic invagination is a rare cause of acute abdomen in the adult population and further, distal colic invagination only represents about 2.1%-9.4 % of colic invagination [4]. Mostly caused by colorectal cancer, rarer causes are described.

The mechanism of intussusception usually involves a pathologic lesion in the bowel wall or mural abnormalities that reduce the normal peristaltism and serves as a lead point, generating an invagination of one segment of the bowel into the other [5].

Typically small intestine intussusception is secondary to benign lesions such as benign neoplasms, Meckel's diverticulum, inflammatory lesions and postoperative adhesions and in only 25% it is due to malignant lesions [6]. On the other hand, large bowel intussusception is more commonly caused by malignant lesions which represents up to 66% of the cases [7].

Abdominal CT scan is the most specific diagnostic test for intussusception and is superior to ultrasonography and endoscopy [8].

Our case then represents the typical presentation of distal colic intussusception.

The histopathologic examination shows that the tumor invades the muscularis. In childhood, it's well known that common ileocaecal invagination may be caused by a disruption of the small bowel peristaltic. We bet that the same phenomena could occur in our case and more generally colic invagination. Indeed, even if the colon is devoid of peristaltis, colonic muscular activity is a key of bowel movement and is affected in common diseases as spastic colon. We haven't found any proof of this assessment in the literature. It could be interesting to proceed a review of histopathologic examinations of different cases of adult bowel invagination to find out if muscularis extension is a prerequisite to intussusception.

## Patient consent statement

The images included in this document are anonymous and do not allow any identification of the patient.
